# Medico-Chirurgical Transactions, Published by the Royal Medical and Chirurgical Society of London

**Published:** 1840-04

**Authors:** 


*Medico-Chirurgical Transactions, published by the Royal Medical and Chirurgical Society of London.* Second Series. Vol. IV.—*London*, 1839. 8vo, pp. 334.

1. The present volume commences with some "Cases of Spasmodic Disease accompanying Affections of the Pericardium. By Richard Bright, M.D." The attention of the profession has been recently called, and we believe with advantage, to a local affection, by some regarded as of a spasmodic, by others as of a paralytic character, depending on unnatural compression or irritation of distant nerves. We refer to the pathology of Laryngismus Stridulus, as explained by the late Dr. Lee. In the present paper, by Dr. Bright, it is attempted to show that various affections of a spasmodic character may be brought on by the effects of inflammation of the pericardium.

"All the cases will be found to agree in the great extent to which certain portions of the nervous system were involved, the irritation of which might, in two of them at least, be looked upon as the cause of death, and in the third bore no small part in the exhaustion of the patient. In each of the cases, the pericardium was importantly implicated; and there was reason to think that the phrenic nerve was the more immediate means of communicating the irritation to the spinal cord. The nervous affection has been in each case of a spasmodic character, but has presented such a variety of aspects, as, on that account, to afford interesting matter for reflection. In one case it was *chorea;* in a second it was *trismus,* terminating in *epileptic convulsion;* and in a third case, modified by the sex of the patient, it assumed more the appearance of *hysteria.*" (p. 2.)

The following is an outline of the first case related by Dr. Bright: A young man, aged seventeen, suffered from general but not acute rheumatic symptoms. When these were subsiding, peculiar spasmodic symptoms occurred, which eventually resembled chorea of most unusual violence. Notwithstanding all the remedies employed, the chorea became worse, and the spasms put on the character of the most violent convulsions. There was some wandering of mind, and personal restraint became necessary. He died about the sixteenth day after the commencement of the spasmodic disease. The body was examined on the following day: the heart, pericardium, and contiguous parts of the lungs were almost exclusively the seat of disease; the heart was adhering to the free pericardium, by the most profuse effusion of firm semi-transparent gelatinous fibrine; this was particularly the case about the base of the heart; the edges of the lung which lay on the pericardium were slightly adherent to that membrane by inflammation; the brain and abdominal viscera were most carefully and minutely examined, and all found perfectly healthy.

The second case was that of a man who, after some imprudent exposure to cold, was seized with pains apparently of a rheumatic character. To these succeeded orthopnœa, quick, irregular pulse, and difficulty in swallowing. The last symptom became very distressing, and was accompanied with much difficulty in opening the mouth; the patient could swallow only with a kind of convulsive catch; the heart was acting very rapidly and spasmodically; the tetanic symptoms rapidly increased, and the teeth became completely closed; the patient could not swallow his saliva, and there were some slight indications of spasmodic action of the muscles of the back. In spite of all remedies, there was no relaxation of spasm; but to this were added severe convulsive attacks, of an epileptic character. After suffering several of these, he died within twenty hours of the first appearance of dysphagia, on which the trismus and convulsions had rapidly followed. After death, the lungs were found to be somewhat gorged with blood, but crepitant throughout almost their whole substance. The lower half of the right pleura, as it covers the ribs and extends over the whole diaphragm, was very much inflamed, and covered with a thin coating of fibrine. The inflammation was still more marked as it ran up towards the root of the lung, and covered the right side of the pericardium, where the phrenic nerve was seen winding its way down the membrane in the midst of indications of the most intense inflammation; and as it ran over the diaphragm, was covered with shreds of recent false membrane. The left pleura was also inflamed. No other morbid appearances were found. *The head and spine were not examined.* Dr. Bright especially calls attention to the combination, in the above cases, of inflammatory affections with convulsive diseases, masking each other; and adds, that, "the great and important point is, to impress upon our minds the fact, that the most violent attacks of spasmodic disease will occasionally owe their existence to inflammation of that portion of the pleura and pericardium where inflammation is often with difficulty detected; that part more particularly where the phrenic nerve in its course or its distribution is to be found." (p. 9.) Dr. Bright has been for many years persuaded of the connexion between chorea and inflammation of the pericardium—a connexion not much attended to, although the combination of the spasmodic disease with rheumatism has long been recognized. It would appear that when disease of a spasmodic character has appeared in connexion with rheumatism, it has been commonly attributed to metastasis of inflammation to the spinal cord. Dr. Bright's attention has been long called to the subject, and he has been long convinced that inflammation of the pericardium has been one among the numerous causes of chorea. Speaking of the connexion of chorea and rheumatism, Dr. Bright says:

"The instances are numerous; and though I doubt not the coverings of the cerebro-spinal mass may be and are implicated, yet I believe that the much more frequent cause of chorea, in conjunction with rheumatism, is the inflammation of the pericardium, and that the irritation is communicated thence probably to the spine, just as the irritation of other parts, as of the bowels, the gums, or the uterus, is communicated, and produces the same diseases." (p. 15.)

We have given Dr. Bright's communication, but not without a strong feeling that there is something very unsatisfactory in its pathology. Every one is conversant with cases of pericarditis and of rheumatic pericarditis, without the accompaniment of any spasmodic disease; and every day's experience brings before our notice the effects of inflammation among parts plentifully endowed with nerves, capable, we may fairly presume, of conveying such a stimulus to the spine as might show itself by convulsion or spasmodic actions. But what is the truth? We do not make the objection with any idea of being able to satisfy it; but it is well when we are disposed to imagine that tangible morbid anatomy gives us a clear insight into pathology, to point out its present deficiencies, that we may not remain content with such partial knowledge.

II. A case of malignant disease of the tongue, removed by ligatures applied beneath the jaw, between the mylo-hyoid, genio-hyoid, and genio-glossi muscles, is related by Mr. Arnott. The result of the operation was recovery. It is useless to abridge the account of the operation, and we cannot give it entire. Those who may be called on to remove diseased parts implicating a large portion of the tongue, will refer with advantage to the description of Mr. Arnott's operation.

III. The "Memoir on Tuphlo-enteritis, by Dr. Burne," will be noticed in another place.

IV. Mr. Salter has related a case of *carditis*, the form of which, as described by him, is undoubtedly rare. But it is probably wrong to conclude that inflammation of the substance of the heart is very rare, although inflammation terminating in the formation of pus may be of unfrequent occurrence. Various reasons might probably be given why, even supposing the muscular substance of the heart to be inflamed, the action should not continue so long as to end in suppuration.

There are probably, says Dr. Hope, cases which, though not attended with effusion of pus, will come under the denomination of universal carditis. Softening and induration are the results of inflammation in other muscles, and analogy points out that they have the same origin in the heart. And further evidence is derived from the fact, that, in cases of pericarditis, the characters in question only occupy a certain depth of the exterior surface of the organ, whence the presumption is almost positive, that they originate in an extension of the inflammation from the pericardium. The morbid appearances discovered after death in Mr. Salter's case were as follows:

"The capillary and other vessels on the bag of the pericardium were distended with red blood. . . . . That portion of the membrane investing the substance of the heart showed also, by its unusual vascularity, striking evidence of its having suffered from inflammation. . . . . The part of the pericardium which had sustained the most intense muscular disturbance was that which lies on and is attached to the diaphragm. Here there was not only a distended state of the capillary and larger vessels, but numerous ecchymosed spots and blotches resembling those observed on the skin in purpura hæmorrhagica. There was no lymphatic, serous, or purulent effusion into the pericardial sac. The heart was somewhat larger than natural, its substance of moderate firmness; large coagula were found in all the cavities. . . . . There were no signs of disease in the lining membrane of the heart or valves. In the ascending aorta there were the appearances of commencing ossification. . . . . Excepting a small portion, of a few lines in thickness, on either surface, the left ventricle had entirely lost its muscular colour; it was of a lightish yellow hue, but still preserving the fibrous character of muscle. From all the cut surfaces of the various sections which were made, could be scraped purulent matter. In some parts absorption had taken place, leaving small cavities in the muscular substance, varying from the size of a pin's head to that of a small pea; these were all filled with pus." (pp. 77-8.)

As will be supposed, there were no symptoms which could be considered as pathognomonic of the above condition. It would appear to be one of unmixed carditis; for we can scarcely agree with Mr. Salter in regarding as of an inflammatory character the vascularity which he has described as affecting the pericardium. Experience has, we believe, shown, that in the absence of some of its effects, such as suppuration, the formation of lymph, &c., it is never safe to infer that inflammation has existed, however great may be the vascularity of diseased parts. And in this case there appears to have been a layer of healthy muscular substance intermediate to the pericardium and deposits of pus.

Although not strictly belonging to the present subject, we cannot forbear to notice that we have lately witnessed an instance of gangrene of the heart. The case was that of a young man, who died of gangrene of the pleura and substance of one lung. In the muscular substance of the heart was a portion of gangrenous matter about the size of a common nut. Of the nature of this morbid condition we entertain no doubt; and it is only recorded here, because the disease, if ever witnessed, appears to be one of infinite rarity. We should add, that the action ending in gangrene in this instance could scarcely be regarded as inflammatory, as the dead portion was immediately surrounded by healthy muscular substance. Mr. Salter's case is one of considerable interest.

v. Some "Notices of the Effects of Lead upon the System; by James Alderson, M.A. and M.D.," follow. If an individual with any partial paralysis is removed away from the causes, predisposing and exciting, of such paralysis, is put upon a generous diet instead of one of an opposite quality, is subjected to a course of medicines of which opiates and quinine form a part, and is required in every way to give rest to the part paralyzed; to what would the majority be disposed to attribute the cure which might take place? And if, rejecting the idea that the cure had been brought about by the combined means, any *one* was to be selected as that to which most benefit was to be attributed, which of the above would be chosen? Dr. Alderson meets with cases of amaurosis as an effect of lead. He takes his patient away from the atmosphere of lead in which she contracted the disease, puts her upon a generous diet, soothes her system with opium and tonics, and ties a handkerchief over her eyes; and then writes a paper to attribute the cure to the effect of preserving the retina from the stimulus of light. We have no doubt of the importance of the means suggested by Dr. Alderson, and cannot but much commend its ingenuity; but we think that too much is attributed to one of the many causes of recovery. The cases are of amaurosis, contracted by lead-workers. As Dr. Alderson's remarks are interesting, we shall quote them:

"The more usual seat of paralysis from lead, which is apparent to observation, is well known to be in the nerves supplying the flexors and extensors of the wrists and arms, especially the extensors; and the plan adopted by Dr. Pemberton, of supplying splints on which to keep the hands and fingers extended, by which the continued extension of the extensor muscles is relieved, and their suspended power recovered, has often been tried with success, both alone and in combination with internal remedies. It is upon the same principle that I have adopted a plan to restore the lost power of the nerves of vision. The action of light is the proper stimulus of the retina, and its continued action, whilst the nervous expansion is paralyzed, is precisely similar to the continued extension of the extensor muscles of the arm and hand; as in this latter case, relieving the muscles from their continued extension, by means of splints, has been found to restore their suspended power, so it was, by analogy, to be supposed, that by removing the stimulus of light altogether from the eye, the power of vision would probably be regained. With this view, a bandage was directed to be applied over the eyes, and constantly worn day and night, so as to preclude the possibility of any light acting upon the nerves of vision. The practice was followed in both cases with success. We see a precisely similar effect arising from the application of the same principle in an over-distended bladder. The over-distension takes away the power of contraction; whilst, by emptying the viscus of its contents, or in some cases only by keeping it empty, the contractile power of the organ is restored." (pp. 82-4.)

The application of this treatment, combined as already mentioned, was followed by success in two cases of amaurosis in lead-workers.

vi. The valuable account of the "Occurrences at the Smallpox Hospital, London, during the year 1838; by Dr. Gregory," it would be a pity to abridge; and we regret that, unabridged, we cannot spare space for it.

VII., VIII. Mr. South has recorded a "Case of Fracture of the Coracoid Process of the Scapula, with Partial Dislocation of the Humerus forwards, and Fracture of the Acromion Process of the Clavicle." Mr. South publishes it as an accident, the occurrence of which has been doubted, and a dissection of which has not, so far at least as he is aware, been hitherto made. We must refer to the volume itself for the description of this accident, as well as for Dr. George Budd's "Statistical Account of Cholera in the Seamen's Hospital, in 1832."

IX. Mr. George Busk, Surgeon to the Dreadnought, has tied successfully the common carotid for an aneurismal tumour within the orbit. The case is related.

X. "On the Softening of Coagulated Fibrine. By George Gulliver, F.R.S." There is a great want of accuracy in the employment of the words pus and purulent; and the old means by which it was habitual to decide on the existence of pus are quite inadequate to distinguish it from other known fluids; whilst it was and probably still is doubtful what different unknown fluids are still spoken of as pus. The paper of Mr. Gulliver shows, and we think satisfactorily, that fibrine in a softened state, bearing as it does an external resemblance to pus, has been mistaken for the latter fluid. We shall give a short analysis of Mr. Gulliver's experiments and opinions. Clots of fibrine taken from the heart were carefully subjected to a somewhat elevated temperature; parts of it became softened; the softened portion was subjected to examination.

"It could not have been distinguished from pus, either by its colour and consistency or by the facility with which it was miscible with water; it was not rendered ropy by caustic volatile alkali, and presented no irridescence when pressed between plates of glass before a candle in the manner pointed out by Dr. Young. Subjected to the microscope, the fluid was found to be loaded with particles extremely variable in size and form; some appeared very dense, others were flaky and larger than pus globules, but the greater number were smaller, extremely irregular in shape, not at all globular like pus, but consisting of very minute granular masses, and a few rounded particles about half the diameter of those of the blood were observed On the addition of acetic acid, all the particles soon disappeared, except a few which seemed more compact, and required a longer time for solution. Treated with sulphurous acid, they swelled considerably, and became nearly invisible." (pp. 138-9.)

"Some of the fibrinous concretion formed, in consequence of inflammation, on the pleura of a dog, was confined in a glass tube, and subjected to a blood heat for eighty-four hours, when it was found to be throughout softened, and presenting just the colour and consistency of pus, from which, however, it was easily distinguishable by the characters noticed in the other trials." (p. 141.)

Fibrine, softened and presenting the characteristics above mentioned, was taken from the femoral and other veins. In most of these cases, Mr. Gulliver states that he has not found any traces of inflammation in the coats of the vessels, such as roughness of the internal membrane, from adherent coagulable lymph, or increased vascularity or suppuration in their cellular sheaths. On the subject of these coagula in veins, it is observed:

"Although the occasional obstruction of the veins by sanguineous coagula, whether softened or not, has become of late years well known . . . . . my observations lead me to believe that the affection is not only more frequent than is generally supposed, but more concerned in disease, a circumstance to which my attention was first directed by Dr. John Davy. Those who have not been in the habit, when examining dead bodies, of accurately attending to this point, would be surprised at the numerous examples of this condition noted in the register of dissections made under his superintendence at Chatham. The clots were in most instances partially whitened, and softened in the centre; they were found in the heart, and also adhering to the inner membrane of the aorta; in the veins of the lower limbs, as well as in the cava, portal, and renal veins; in the pulmonary vessels, and in the sinuses of the dura mater. They were chiefly observed in subjects who, having long suffered from various chronic maladies, had great prostration of the vital powers, and languor of circulation in the last days of existence..... If, therefore, a vein becomes obstructed with coagulated blood or fibrine which cannot easily be absorbed or organized, in consequence of the very feeble vitality of the patient, the clot may be expected to soften, and it is accordingly in such cases that the affection may be often found after death, although never suspected during life." (pp. 147-9.)

The softening of coagulated fibrine is regarded as distinct from suppuration, and as constituting a considerable proportion of the cases generally denominated suppurative phlebitis. Pus is sometimes observed, mixed with the softened fibrinous matter:

"In two instances, some purulent globules were observed, completely insulated, in the centre of a clot, from the neighbouring tissues. But in both these cases pus was also detected in the blood; and when it is considered how frequently this contamination exists, it will not appear surprising that the particles of pus should become entangled in coagula contained in the vessels. Nor does this circumstance at all invalidate the conclusion that the softening of coagulated fibrine is a disease of much interest and importance, quite distinct in its nature from suppuration, although the two affections have been generally regarded as identical." (p. 152.)

xi. "Contributions to the Pathology of the Spinal Cord; by William Budd, m.d.," constitute the next communication. These contributions consist of several remarkable cases, with observations thereon, illustrative of various points of the pathology of the spine, to which attention has of late been much directed. One of Dr. Budd's objects has been to show, from his cases, that the involuntary movements excited in the palsied limbs were the same in kind as those observed in decapitated animals. The cases are well deserving of attentive perusal. In the first case there was total loss of voluntary power in the lower extremities, and involuntary muscular contractions were excited by applying stimulus to the skin or mucous membranes. This case, and others to which we shall allude, are of interest, as illustrating the probable want of connexion of the excito-motory actions with sensation:

"Although sensation was never quite extinct in Larg's lower extremities, it was evidently not concerned in the production of the involuntary contractions; for when an artificial mode of stimulus was employed, the convulsions were uniformly the most vigorous in that case in which the application was not felt by the patient, namely, when the hollow of the foot was lightly touched with a feather. Here, then, we have oral evidence from the subject of experiment, that these acts are quite independent of sensation and volition. The fact that such vigorous convulsions were excited by touching the hollow of the foot with a feather is of great interest, and tends to show that the nerves which transmit these impressions to the spinal marrow are—if not the same with those of true sensation—most actively excited by a stimulus which produces strong impressions on the sensitive nerves. Every one knows how urgent is the sensation of tickling in the hollow of the foot. Volkmann has proved that these two kinds of nerves (if there be two kinds) arrive at the spinal marrow in one bundle, namely, the posterior roots. That their distribution should be alike is obvious, for their purpose is the same—the reception of external impressions. We must not fail to remark, that, in the case of Larg, convulsions were never induced by excitement of the passions." (pp. 159-60.)

Respecting similar phenomena in another case, it is remarked of the convulsions :

"Their independence of sensation was curiously shown by the difference of effect from the application of a heated plate, and of the same plate at common temperature, although the difference of heat in the two cases was not felt by the patient, the sense of contact being all that was perceived. This continuance of the sense of contact, while that of temperature was completely lost, is a novel and interesting illustration of the speciality of sensations; a subject so ably treated by Sir C. Bell." (p. 175.)

Without being able to give it any satisfactory reply, Dr. Budd moots the question as to the cause of the susceptibility to involuntary contractions in muscles which do not commonly exhibit it, when in a paralytic state,—whether this is to be ascribed to the withdrawal of the influence of volition, or to an irritation of the cord, and consequent increase of its motor power. Dr. Budd's opinion is in favour of the cooperation of both causes in the majority of instances, especially where the involuntary contractions are powerful and extensive.

The remarks of Dr. Hall in the next communication bear upon the subject, as well as upon other facts in Dr. Budd's paper. The convulsions observed were not associated with a sense of fatigue, but the power that caused them was subject to exhaustion, and removed by rest. "Strychnia given internally increased the susceptibility to involuntary movements, and caused spontaneous convulsions that were limited to the palsied parts."

XII. The next communication is entitled "Memoirs on some Principles of Pathology in the Nervous System. By Dr. Marshall Hall." The subject of the paper is the *condition of muscular irritability in paralytic limbs.* On this subject discrepant opinions exist; some maintaining that the irritability of muscular fibre remains in paralytic limbs; others the contrary. The object of the author is to explain this difference of opinion, by showing that previous physiologists have taken a partial and incorrect view of the matter. It is essential to distinguish between cerebral paralysis, or that which removes the influence of the brain, and spinal paralysis, or that which removes the influence of the spinal marrow. We have not space to devote to the experiments and opinions quoted from Prochaska, Nysten, Legallois, &c., by Dr. Hall, to establish the statement above made respecting the supposed conditions of muscular irritability in paralytic limbs, nor indeed is it necessary; but we feel pleasure in laying before our readers, with some minuteness, the author's mode of reconciling the differences alluded to.

If strychnia be given to a paralytic, it is *often* the paralytic limb which is first affected by it. Is the irritability of the muscular fibre thus affected increased? The affirmation of this question, says Dr.

Hall, would explain the phenomenon. The following experiments, in illustration of this point, are quoted: "A little child, aged two years, was perfectly paralytic of the left arm. The slightest shock of galvanism was directed to be applied which could produce an obvious effect. It was uniformly observed that the paralytic limb was agitated with a degree of energy which produced no effect on the healthy limb." (p. 200.) In similar cases, and in hemiplegic cases, the experiment was repeated, and, with two exceptions, the results were the same, the paralytic limb being always more shaken than any other part. Dr. Hall next experimented upon animals. The experiments were made on six frogs.

"I divided the spinal marrow immediately below the origin of the brachial plexus, and I removed a portion of the ischiatic nerve of the right posterior extremity. I had immediately, or more remotely, the following interesting phenomena: 1st. The anterior extremities alone were moved spontaneously. . . . 2d. Although perfectly paralytic, in regard to spontaneous motion, the left posterior extremity, that still in connexion with the spinal marrow, moved very energetically, when stimulated by pinching the toes with the forceps. 3d. The right posterior extremity, or that of which the ischiatic nerve was divided, was entirely paralytic, both in reference to spontaneous and excited motions. 4. After the lapse of several weeks, whilst the muscular irritability of the left posterior extremity was gradually augmented, that of the right was gradually diminished. . . . . In this interesting experiment, we have, then, first the phenomena of loss of spontaneous motion on removing the influence of the brain, the excited or reflex actions remaining, and the loss of these on removing the influence of the spinal marrow; secondly, in the case of mere cerebral paralysis, we have augmented irritability, and in that of the spinal marrow, we have the gradual diminution of this property. 5. Strychnine being now administered, the anterior extremities and the left posterior extremity, or that still in connexion with the spinal marrow, became affected with tetanus; but the right posterior extremity, or that severed from all nervous connexion with the spinal marrow, remained perfectly flaccid. 6th. Lastly, the difference in the degree of irritability, in the muscular fibre of the two limbs, was observed, when these were entirely separated from the rest of the animal. In a word, the muscles of the limb, paralyzed by its separation from both cerebrum and spinal marrow, had lost their irritability; whilst those of the limb, separated from its connexion with the cerebrum only, but left in its connexion with the spinal marrow, not only retained their irritability, but probably possessed it in an augmented degree. The next question came to be—Do these phenomena obtain in the human frame? I visited a patient affected with hemiplegia, including paralysis of the face, and I passed a slight galvanic shock through two pieces of metal, of which one was placed over each cheek. The muscles of the paralytic side were most affected. I repeated the experiment with the same result. I now compared with these, two cases of injury of the facial nerve, passing the galvanic shock in the same manner, through the fibres of the orbicularis: it was now the muscle of the healthy side which was affected by the galvanism, the eyelid of that side being closed, whilst that of the paralytic side gaped as before. I next compared the effect of galvanism in two cases of complete paralysis of the arm, one hemiplegic, the other the result of dislocation of the shoulder. The muscles of the former were more, those of the latter less irritable than those of the healthy arm, respectively, as were also those of the arm of a patient affected with the paralysis induced by lead. Lastly, I compared the cases of paralysis of the lower extremities, one arising after pertussis, and therefore cerebral, the other, I think, from disease within the lumbar vertebræ; in the former there was augmented, in the latter diminished irritability. By means of these experiments and observations, we are enabled, I believe, to explain all the apparent discrepancies between the statements of former authors and between each of them and my own. . . . . We may conclude that, in cerebral paralysis, the irritability of the muscular fibre becomes augmented, from want of the application of the stimulus of volition; in paralysis, arising from disease of the spinal marrow and its nerves, this irritability is diminished, and at length becomes extinct, from its source being cut off." (p. 201, et seq.)

The same facts may be applied to the diagnosis between spinal and cerebral paralysis.

We subjoin the conclusions at which Dr. Hall arrives from the foregoing and other observations:

"1st. That the spinal marrow, exclusive of the cerebrum, is the source of the muscular irritability.

"2d. That the cerebrum is, in its acts of volition, an exhauster of that irritability.

"3d. That, in muscles separated from their nervous connexion with the brain, we have augmented irritability.

"4th. That, in muscles separated from their nervous connexion with the spinal marrow, we have, on the contrary, diminished irritability.

"5th. That the degree of the irritability of the muscular fibre of paralytic limbs, compared with that of the muscles of the healthy limbs, will afford us a source of diagnosis between cerebral and spinal paralysis, and especially between

{ "1, Hemiplegia of the face, and
{ "2, Paralysis of the facial nerve;

{ "3, Hemiplegia of the arm or leg, and
{ "4, Disease of the nerves of these limbs;

{ "5, Disease of the spinal marrow in the dorsal region, and
{ "6. Disease of the cauda equina in the lumbar region, &c.

"6th. That the greater influence of emotion, of certain respiratory acts, of the principle of tone, &c., on the muscles of certain paralytic limbs, than on those of healthy limbs, depends on their augmented irritability.

"7th. That the same principle explains the greater susceptibility of the muscles, in certain cases of paralytic limbs, to the influence of strychnine.

"8th. That, in the conclusions of M. Fouquier, Professor Müller, &c., a sufficient distinction was not made between the influence of the cerebrum and of the spinal marrow, which in this, as in so many other respects, have such different properties.

"9th. From these, and other experiments and observations, I conclude, too, that sleep restores the irritability of the muscular system, by arresting the acts of volition, which exhaust or diminish it; muscular efforts, on the other hand, diminish the irritability and induce fatigue."

XIII. Mr. Stafford has described a "Case of Enlargement from Melanoid Tumour of the Prostate Gland, in a Child of five years of age."

XIV. Mr. Curling gives an instance of "Congenital Absence of Pericardium," witnessed by himself, together with the few similar cases recorded in works of morbid anatomy.

XV. The next paper, communicated by Mr. Cæsar Hawkins, is "On a Peculiar Form of Congenital Tumour of the Neck." The kind of tumour here described is "composed of many cysts joined together, in which the proportion of organized matter is so considerable, as to give a more solid character to the tumour, and make it deserve the name of cystic tumour, as much as the apparently analogous cases of cystic sarcoma, occasionally found in the breast, testis, or ovary of adults." (p. 232.) Mr. Hawkins has met with seven such tumours in the necks of young children. Two of these cases are detailed, and a minute description of the morbid anatomy in one of the cases is given. It is observed that the numerous cysts, composing the tumour, are formed in the common cellular tissue—that they occur more often in the neck than elsewhere. The diagnosis is confessedly obscure. "When [the component cysts are] numerous, and full of liquid, and small in size, they feel like enlarged glands, or other solid globular bodies; when numerous, and only half filled, they become soft and compressible; but in both these cases, the existence of fluid is difficult to be detected, in comparison with those cases in which only a few larger cysts exist. The resemblance to fatty tumours is considerable, and the deception is assisted by the quantity of fat generally situated beneath the skin, and filling up the inequalities of the surface of the tumour. They are also much like a subcutaneous nævus, which is so often developed in the same situation, when the cysts are half filled. . . . . . In each case that I have seen, however, the existence of globular bodies, in some of which fluid was perceptible, distinguished the tumour from every other kind likely to be formed in infancy." (p. 240.)

The inaccessible situation and intricate connexions of the deeper parts of these tumours, in many instances, render the endeavour to extirpate them most unjustifiable. Their treatment may be as follows: 1. Evacuating the cysts by a grooved needle from time to time. 2. Pressure, when the situation of the tumour does not forbid it. 3. Stimulant applications. Respecting the last, Mr. Hawkins observes, "judging from the few cases I have seen, their effect would appear to extend to some depth below the skin, and to act both on the cysts and their fluid contents." We have never met with any of the cases described in this paper; but if we may draw any inferences from the treatment of other forms of encysted tumours, we should be disposed to employ setons in these cases, especially where the cysts are few in number, and of any size. Mr. Hawkins does not allude to this mode of treatment, and many surgeons appear to have an objection to the employment of setons, which we believe to be groundless. Our own mode of using them in analogous cases, is what we should be disposed to try in these, i. e., to introduce one or two threads at first; if too much irritation or inflammation was induced by these, to withdraw one or both, and wait for a more convenient opportunity; if sufficient action did not follow their introduction, to add threads until our object was obtained. The objection which attaches itself to setons, we believe to depend on their being employed, when the state of the health is not such as to justify their employment—on the want of care, to produce the least degree of action, which is consistent with the end which we have in view,—and in neglect of general and local management whilst that action is in progress. Being guided by proper principles, we think that setons might be effectually employed, so as to dissipate, at least, a portion of the tumours described by Mr. Hawkins.

XVI. Two Cases of "Measles occurring oftener than once in the same

Individual" are detailed by Dr. John Webster. The cases must be regarded as authentic, and in that respect valuable.

XVII. Mr. Solly has recorded a "Remarkable Case of Dry Gangrene, occurring in a Child three years and seven months old." The age of the child, and the extensive ravages of the gangrene, render this a remarkably interesting case in many points of view, but we can but refer to it in this place.

XVIII. Mr. Lever has communicated "Statistical Notices of 120 Cases of Carcinoma Uteri," including under the term only such cases as all agree in terming "Carcinoma." Before commencing his investigation, Mr. Lever states that he held many opinions respecting the disease, which were contradicted by his investigations conducted on the numerical plan. This would appear to be a tolerably certain effect of numerical investigations, and certainly is a fact of great importance. The period of life most obnoxious to the disease, appears to be from the fortieth to the fiftieth year. Mr. Lever's tables "afford a complete refutation to the statement that celibacy favours the development of the disease.". . .

"Only 25 women, or 20·8 per cent., had enjoyed good uterine health in early life, while 95, or 79·16 per cent., had suffered either from (what is termed) functional disease, or syphilis. The most frequent malady was dysmenorrhœa, with which no less than 66 had been afflicted. The proportions stand thus:—

| | | per cent. |
|---|---|---|
| Those who had good uterine health | | 20·8 |
| Those who had suffered from | Amenorrhœa | 15·8 |
| | Vicarious Menstruation | 0·83 |
| | Menorrhagia | 1·66 |
| | Dysmenorrhœa | 54·16 |
| | Syphilis | 6·6" |

(p. 271.)

XIX. Mr. Curling has described, under the name of "Dactylius Aculeatus," an entozoon, many of which were voided from the urethra of a little girl. The generic and specific descriptions are as follows:

"Genus DACTYLIUS (from δακτύλιος, annulus). Corpus teres elasticum annulatum et utrinque attenuatum, caput obtusum os orbiculare, anus trilabiatus.

"DACTYLIUS ACULEATUS. Capite obtuso, toto corpore aculeorum serie multiplici armato, caudâ obtusâ et annulatâ.

"Hab. in Hominis vesicâ urinariâ" (p. 282-3.)

There were no disturbances of the urinary organs corresponding with the presence of these entozoa. Mr. Curling considers that parasitic animals occur in the human body far more frequently than is generally supposed, being commonly overlooked from the minuteness of their size. He subjoins, on the same point, his opinion, that the trichocephalus dispar would probably be found in the large intestines of most bodies after death. "During the last winter," it is added, "they were detected in most of the bodies examined at the London Hospital; indeed, in nearly all the cases, in which much pains were taken in looking for them in the intestinal canal of healthy persons destroyed by severe injuries, as well as of those cut off by acute and chronic diseases." (p. 285.) These are interesting facts, but a question still remains, whether the animals, found in the intestinal canal after death, had existed there as such during the life of the individual whose corpse they are found to inhabit.

xx. Mr. Liston has made some "Remarks on the Acute Form of Anasarcous Tumour of the Scrotum."

"This distension [of the scrotum] is or is not attended by redness or erythema of the surface; but there is reason to think, from the suddenness of the accession, and from the appearances on exposing the cellular tissue, that there is no actual inflammation of its texture; there being no induration, nor any appearance of lymph or puriform fluid in the areolæ. The affection has generally supervened upon abscess or ulcer, perhaps trifling, in the perineum or groin. Its accession has been sudden, the swelling and tension becoming very great and alarming, even within a few hours. The most dependent part, generally the posterior, will be found at a very early period to present one or more deeply-seated ash or tawny-coloured spots; these extend; the integument is speedily involved; and unless active measures be adoped, the entire coverings and investments of the testicles will be destroyed, and these organs exposed. The progress of the case will depend much upon the state of the patient's system, and the nature of the infiltration. . . . . But in hospital practice, . . . . the surgeon will do well to look with suspicion at any sudden swelling in this region, and be prepared to treat it actively." (pp. 289-90.)

It is necessary to distinguish between these cases and cases of infiltration of urine. Free incisions are requisite in either case, but the urinary passages, in the former cases, do not require to be interfered with. Mr. Liston has related several cases of the affection above described.

xxi. Dr. Lee has given "an Account of a Fœtus of seven months, with its placenta partially adherent to a nævus occupying the scalp and dura mater."

xxii. Then follows an exceedingly interesting communication from Mr. A. Nasmyth, "On the Structure, Physiology, and Pathology of the Persistent Capsular Investments and Pulp of the Tooth." The points to which Mr. Nasmyth particularly wishes to call attention are, "the capsular investment on the surface of the enamel; secondly, the layer external to the crusta petrosa; and, thirdly, the structure and development of the 'fourth constituent substance or ossified pulp." The paper well merits a careful perusal; we should gladly, if it were practicable, do something more than briefly allude to its chief points of importance. With regard to the cellular investment on the surface of the enamel, the author states that it is easy to demonstrate its existence. It will, of course, be most easily found in those teeth which have been the least used.

"The teeth require merely to be macerated in muriatic acid, diluted to one eighth of its ordinary strength, and in the course of a few minutes generally, it will be found to be loosened from the surface; and, as soon as it is once partially separated, it may be easily altogether detached. In all cases where this covering has been removed by means of acid, it has, of course, the appearance of a simple membrane, in consequence of the earthy deposits having been dissolved, and of there being only present the animal tissue. The structure and appearance of the covering, detached in this manner from the enamel, are the same in every respect as those observed in the capsule of the unextruded tooth; consisting, like it, of two layers, fibrous externally, and having on its internal surface, the peculiar reticulated appearance common to both." (p. 313.)

Speaking of the crusta petrosa, concerning which various opinions have been held, the author states it as his opinion, that the capsular investment, above alluded to, has been spoken of as the cementum, or crusta petrosa. The same osseo-membranous covering in the human tooth is much thicker round the fang than round the enamel, and the portion which invests the fang increases in size somewhat in relation to the age of the individual. It is by means of this capsular membrane that Mr. Nasmyth considers that the milk-teeth are absorbed. On this point it is observed:

"The portion of the capsular membrane under consideration seems to be the only agent capable of effecting the process of absorption; and we may have ocular demonstration of its performing this, by withdrawing carefully a deciduous tooth when it is near falling off. The fangs will then be found to have almost disappeared, and only a small portion of the membrane to remain; the latter is observed on the spot from which the tooth has been removed; it is in connexion with the pulp, and the whole is highly vascular, and retains an exact impression of the surface of the tooth which was opposed to it. As absorption can only be carried on by the surface in immediate contact with the part absorbed, it follows that this membrane must be so organized as to be able to effect that process." (p. 319.)

Mr. Nasmyth seems altogether to leave out of consideration the organization of the teeth themselves. Notwithstanding that there are many very high authorities who discredit their organization, there is a class of facts which cannot be explained but by admitting it, and if so the absorption may be effected by the vessels of the tooth itself.

The disease of the teeth termed exostosis is a consequence of a morbid action in this capsular membrane. It is well known that this may take place in a variety of forms. It is stated that these bony growths may sometimes be detected by a slight alteration in the colour of the tooth, and by its yielding a little more than natural in one direction, when forcibly pressed. These conditions, however, exist in so many other states of the teeth, that we cannot regard them as of any diagnostic value. It is far more important, and far too often overlooked, that "cerebral congestion, rheumatism, earach, tic douloureux, &c., are often supposed to exist" when the symptoms so designated are dependent on exostosis of the teeth. The observations on this subject in the valuable work of Mr. Bell cannot be too strongly impressed upon the mind. We hear too of physicians who never undertake the conduct of a case until the teeth have been examined, and the appropriate remedies applied to such as are defective. And although this extreme is unnecessary, it contains, as do all similar extremes, a valuable practical lesson, which we shall do well to profit by. The small notches, sometimes met with on the necks of teeth, and which are acutely painful when touched, are supposed to be effected by the vessels of the capsule covering the fang.

Illustrations are given of the ossified pulp. In some of the lower animals, the pulp appears to be normally converted into an earthy structure, intermediate in appearance between bone and ivory. In man, the conversion of the pulpy membrane into osseous matter is either partial or general. It is of frequent occurrence, often coming on after the actual or other cautery has been used to allay sensibility without actual destruction of the internal membrane. The formation of this substance is always attended with a peculiar uneasiness of the tooth itself and neighbouring parts; but the patient is often not relieved "till the malady, having crept on to the external capsular membrane, affords to the careful observer a decided demonstration of its existence." Mr. Nasmyth promises the society further communications on the structure of the teeth, which, if they equal the present, must be regarded as very valuable.

XXIII. The volume terminates by some remarks "On the Structure of the Corpus Luteum, by Robert Lee, M.D." Dr. Lee has examined several specimens of corpus luteum, and believes that its structure is different from that which has hitherto been described. With Dr. Montgomery, and all the best authorities, he considers that the *true* corpus luteum is never formed but as a consequence of impregnation. But it is well known that the same name has been given to various bodies in the ovaria which are not, in fact, corpora lutea. And it hence becomes important to determine the true characteristics of these bodies. The two prevailing opinions are: 1, that it is by the thickening of the inner membrane of the Graafian vesicle that the corpus luteum is formed; and 2, that it is formed by, and inclosed between, the two membranous coverings of the vesicle. The latter opinion is that entertained by Dr. Montgomery. Dr. Lee offers another explanation of the structure of this body. He says, having described his observations on the subject, "we may conclude that it is neither produced by a thickening of the inner layer of the Graafian vesicle, nor by a deposit of a new substance between its two coats, but that it is formed around the outer surface of both these coats of the Graafian vesicle, and that the stroma of the ovarium is in immediate contact with the external surface of the yellow matter." In a postscript of this paper, Dr. Lee states that he "examined the ovarium and corpus luteum with Sir Astley Cooper and Mr. Wharton Jones, and the result is, that the correctness of the view which has been taken of the structure of the corpus luteum in this paper, is now put wholly out of doubt."

